# Comparing the Postoperative Analgesic Efficacy of Ketorolac and Tramadol After Open Inguinal Hernia Mesh Repair: A Randomized Controlled Trial

**DOI:** 10.7759/cureus.71363

**Published:** 2024-10-13

**Authors:** Syed M Ahmed, Sidra Shabbir, Nauman A Rana, Atia Khatoon, Umar F Ghani, Irmaghana Basharat, Muhammad N Khan, Fahd M Hameed, Muhammad F Dar

**Affiliations:** 1 General Surgery, Pakistan Air Force Hospital, Islamabad, PAK

**Keywords:** inguinal hernia repair, ketorolac tromethamine, lichtenstein’s repair, tramadol hcl, visual analog scale

## Abstract

Background

Opioids are the mainstay for postoperative pain control. However, due to the increasing dependence on opioids and their side effects, multiple adjuncts are used to reduce opioid consumption, including non-steroidal anti-inflammatory drugs (NSAIDs). Inguinal hernia repair is one of the most common procedures performed by general surgeons worldwide. Despite various advancements in operative techniques, postoperative pain remains a major risk factor for morbidity and delayed hospital discharge following surgery.

Objective

The objective of this study was to compare ketorolac with tramadol for postoperative pain control following open inguinal hernia mesh repair.

Methods

This was a randomized controlled trial. Sixty patients undergoing unilateral primary open inguinal hernia repair with mesh were randomly divided into two groups: Group A received injection ketorolac intravenously postoperatively, while Group B received injection tramadol intravenously following surgery. Pain scores and vitals were measured at two, six, 12, and 24 hours post procedure. Other secondary outcomes included the time for nausea to stop, time to pass flatus, and time to start ambulation. Data was analyzed by independent-sample t-test using IBM SPSS Statistics for Windows, Version 30.0 (Release 2024; IBM Corp., Armonk, New York, United States); a p-value of < 0.05 was considered significant.

Results

Pain scores were lower in the ketorolac group when compared to tramadol. However, patients in the tramadol group had nausea for a longer duration, delayed return of bowel function, and took longer to start ambulation. There was no significant difference in vitals.

Conclusion

Injection ketorolac, when used in isolation, provides more effective postoperative analgesia along with fewer side effects when compared to tramadol in inguinal hernia repair.

## Introduction

Inguinal hernia repair is one of the most common surgeries performed by general surgeons, with more than 800,000 surgeries being performed annually just in the United States [1], and around 20 million surgeries being performed worldwide [2]. Inguinal hernias make up almost 75% of all abdominal wall hernias, showing a male predominance [1]. Over the decades, multiple techniques have emerged for the repair of inguinal hernias; however, in terms of recurrence, tension-free mesh repair is the preferred method of choice, which can be done either open or laparoscopically [3]. Various studies have looked at the ideal method, but the fact that there are so many techniques available means that there is no one best answer. Open tension-free mesh repair is generally the preferred technique for unilateral primary inguinal hernias, especially in resource-limited countries [3].

Despite the advancements in operative techniques, postoperative pain is a major contributing factor to the overall morbidity from surgery. It results in delayed ambulation, delayed return to daily activities, and a cause for delayed discharge from the hospital [4]. The ideal postoperative analgesic regimen should be one that reduces or eliminates pain and discomfort with the fewest side effects and minimal cost [5,6]. Despite decades of advancement in pain management, opioids remain the mainstay of postoperative pain control [7]. Commonly used opioids include morphine, tramadol, fentanyl, and nalbuphine. The major side effect of opioid use includes respiratory depression, which requires respiratory and oxygen saturation monitoring postoperatively. Other common side effects include nausea, vomiting, pruritis, ileus, and constipation [8,9]. Furthermore, the increasing use of opioids causes dependency and has resulted in a devastating and lethal health problem of opioid overdose not only in America but also in Canada, England, and Australia, among other countries [10].

An important technique to decrease the use of opioids is to use alternative medications for their opioid-sparing effects. Non-steroidal anti-inflammatory drugs (NSAIDs) are useful in decreasing opioid use postoperatively, and those commonly used are ketorolac and diclofenac [7]. Ketorolac has been shown to reduce opioid use by as much as 25-45% in colorectal surgeries [11-13].

In our clinical setting, we routinely use only injection ketorolac following open inguinal hernia repairs and have found satisfactory postoperative analgesia. We, therefore, hypothesized that ketorolac is as effective as tramadol in postoperative analgesia, with fewer side effects, when used in isolation following open inguinal hernia repair.

## Materials and methods

This was a double-blind randomized controlled trial conducted at a tertiary care hospital, Pakistan Air Force Hospital, Islamabad, Pakistan, from August 2023 to July 2024. Approval was obtained from the Pakistan Air Force Hospital Ethical Review Committee (approval number: SGR-2021-137-2499-2) and was performed in accordance with the ethical standards laid down in the 1964 Declaration of Helsinki and its later amendments. The trial was registered on clinicaltrials.gov (NCT06608056).

The sample size was calculated using the WHO sample size calculator, in which the level of significance was kept at 5%, and the power of test at 90%. The test value of population mean was 6.65 [14] and the anticipated population mean was 2.74 [15], with a population standard deviation of 1.54 and variance of 2.3716. These values gave a total sample size of 60 subjects for the study.

Patients were either American Society of Anesthesiologists (ASA) categories I or II and aged 18-75 years. Experienced general surgeons performed the procedure on patients who presented with unilateral primary inguinal hernias. Exclusion criteria included patients who presented with incarcerated, strangulated, or recurrent inguinal hernias, had a drain placed intraoperatively, were on chronic pain medications, or had received analgesics 24 hours prior to the surgery. Patients with a body mass index (BMI) greater than 40 or those allergic to the medications being tested were also excluded from the study.

**Figure 1:**
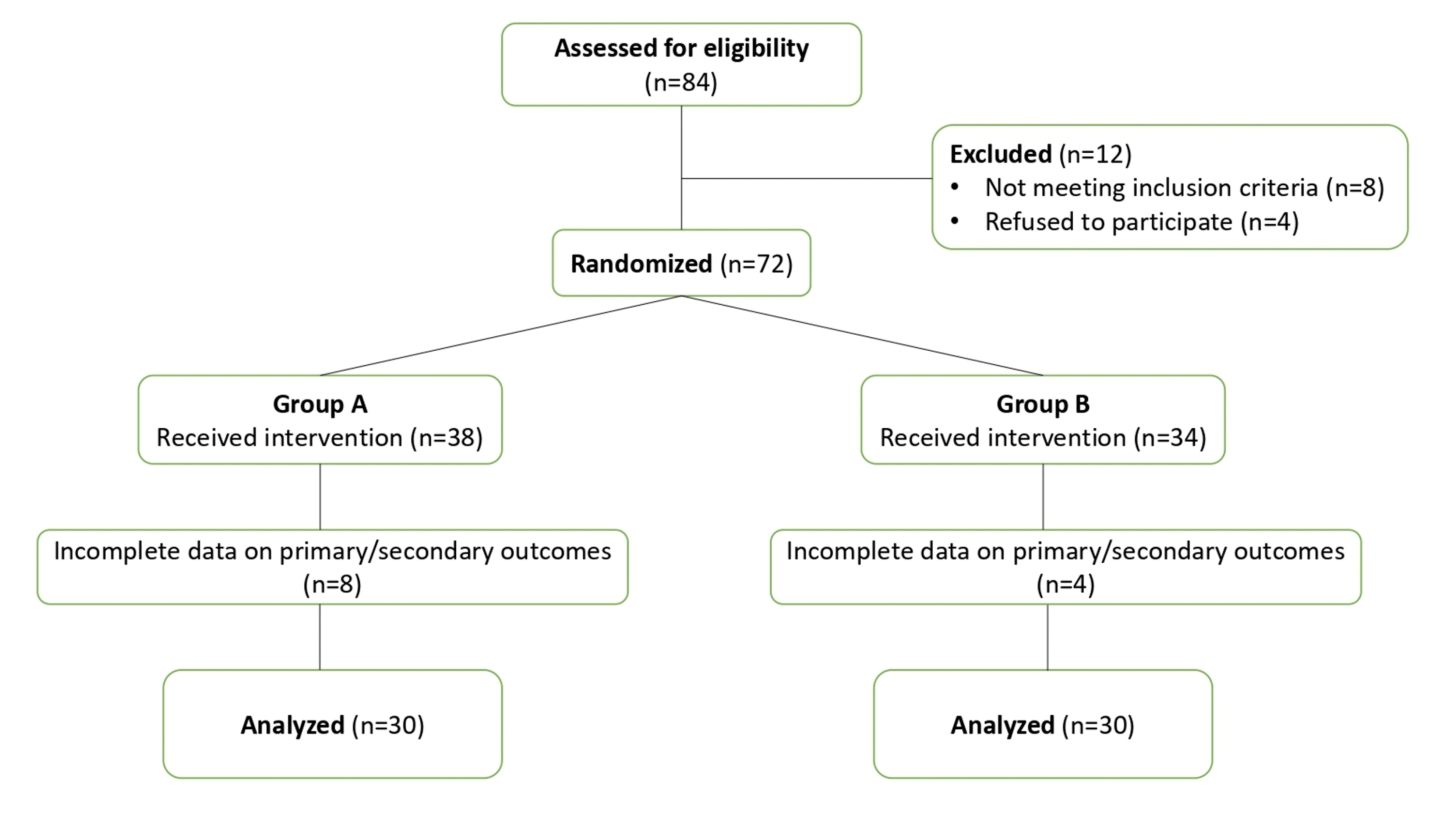
CONSORT flow diagram CONSORT:  Consolidated Standards of Reporting Trials

The Consolidated Standards of Reporting Trials (CONSORT) principles were followed while conducting this study. A total of 84 patients were assessed for eligibility of which 72 patients undergoing open primary unilateral inguinal hernia mesh repair met inclusion criteria and gave informed consent (Figure 1).

All procedures were done under spinal anesthesia. The patients were randomly assigned to two groups depending on the postoperative analgesic used, using simple randomization with computer-generated numbers. In Group A, patients received injection ketorolac (Toradol (ketorolac tromethamine)) 30 mg intravenously every eight hours. In Group B, patients were given injection tramadol (Campex (tramadol hydrochloride)) 50 mg intravenously every eight hours. In both groups, patients received standard antibiotics and proton pump inhibitors (PPIs). House surgeons blinded to the study groups collected postoperative data on a standardized proforma, including a visual analog scale for pain at two, six, 12, and 24 hours post procedure. If the patient’s pain score was more than 5 and not settling with the analgesic being tested in the group, then rescue analgesia (injection nalbuphine 5 mg intravenously) was used, and the timing of administration was noted on the proforma. At each of these time points, the patient’s vitals were also recorded, along with whether the patient was experiencing any nausea or vomiting, if they had started passing flatus, and if they had started ambulating.

Out of the 72 patients randomized to the two groups, a total of 12 patients were not included in the final analysis of data (Figure 1), as their primary/secondary outcomes data was incomplete due to technical issues. So a total of 60 patients were included for analysis; 30 from each group. Statistical analysis was performed using an independent-sample t-test on IBM SPSS Statistics for Windows, Version 30.0 (Release 2024; IBM Corp., Armonk, New York, United States). A p-value of ≤ 0.05 was considered to be significant.

## Results

Demographics

Demographics were similar in both groups, with mean age in Group A (ketorolac) being 56.03 years (Standard error of mean (SEM) ± 2.10) and in Group B (tramadol) being 53.47 years (SEM ± 2.02). There was a male predominance in the subjects, with only one female patient present in both groups.

Postoperative pain

**Figure 2:**
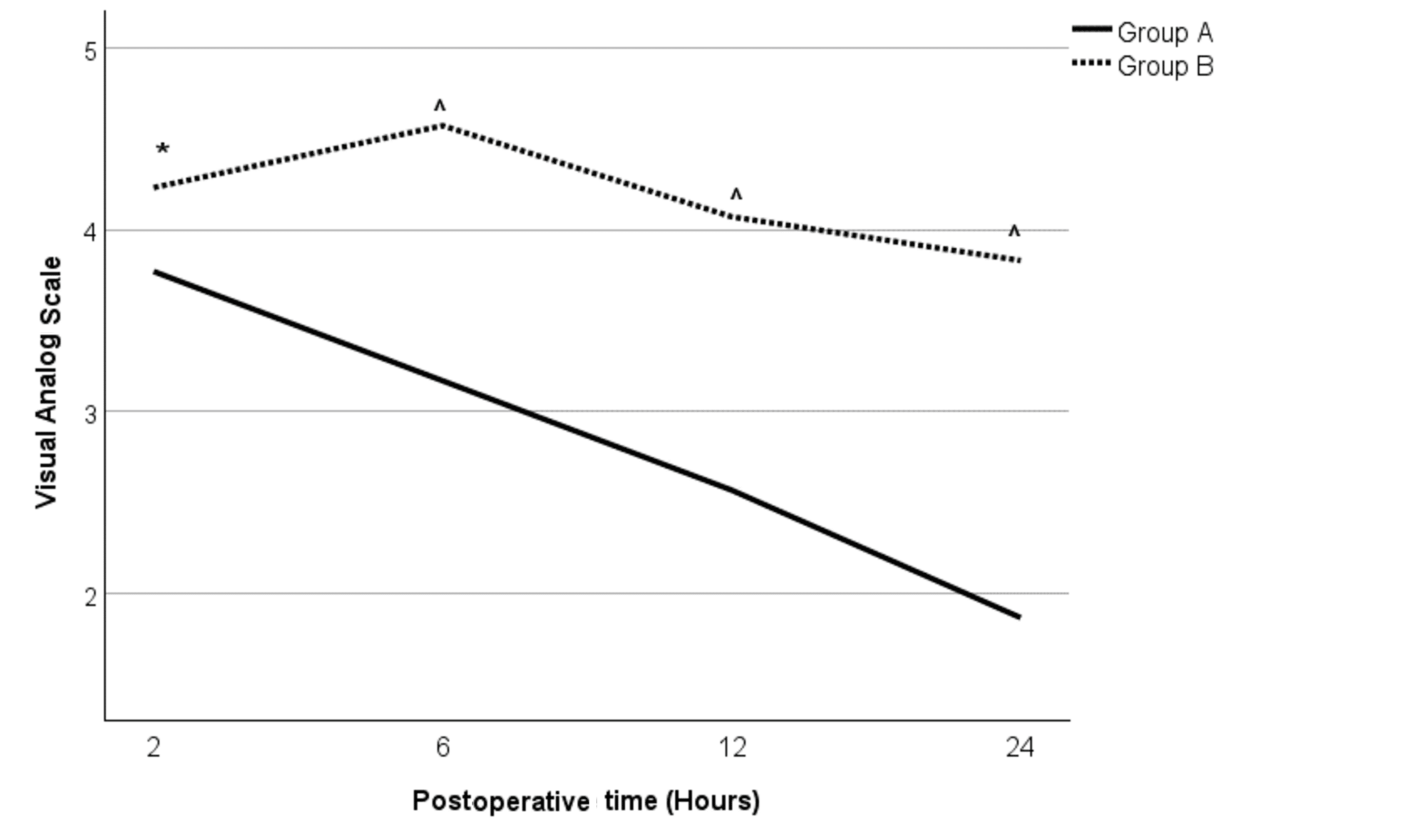
Pain score (as measured by visual analog scale) for Groups A and B Test used: independent-sample t test. p ≤ 0.05 considered significant * p = 0.01 when compared to Group A; ^ p < 0.001 when compared to Group A

When comparing postoperative pain (as measured by the visual analog scale (VAS)), Group A showed a better analgesic effect than Group B, which was statistically significant throughout the study period (Table 1, Figure 2). At two hours following the surgery, patients in Group A had a mean pain score of 3.77 ± 0.13 as compared to 4.23 ± 0.11 in Group B (t = -2.662, df = 58, p = 0.01). At all the other time points, at six, 12, and 24 hours, Group A still showed improved pain scores (3.17±0.15, 2.57±0.14, and 1.87±0.14, respectively) than Group B (4.57±0.15, 4.07±0.14, and 3.83±0.14, respectively) (p<0.001). Even when looking at the rescue analgesic requirements, only one patient required rescue analgesia in Group A (at six hours post procedure), whereas three patients in Group B required rescue analgesia (two at six hours and one at 12 hours postoperatively).

**Table 1:** Statistical analysis of postoperative pain Test used: independent-sample t test. p ≤ 0.05 considered significant VAS: visual analog scale; SEM: standard error of mean

Group	Postoperative time (Hours)	VAS Score (Mean ± SEM)	t	df	p
A	2	3.77±0.13	-2.662	58	0.01
B		4.23±0.11			
A	6	3.17±0.15	-6.568	58	< 0.001
B		4.57±0.15			
A	12	2.57±0.14	-7.454	58	< 0.001
B		4.07±0.14			
A	24	1.87±0.14	-10.002	58	< 0.001
B		3.83±0.14			

Vital signs

**Figure 3:**
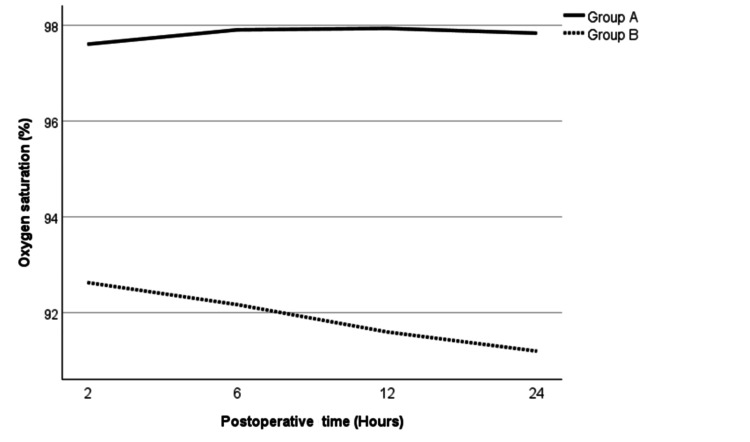
Mean oxygen saturation for Groups A and B Test used: independent-sample t test. p ≤ 0.05 considered significant

**Figure 4:**
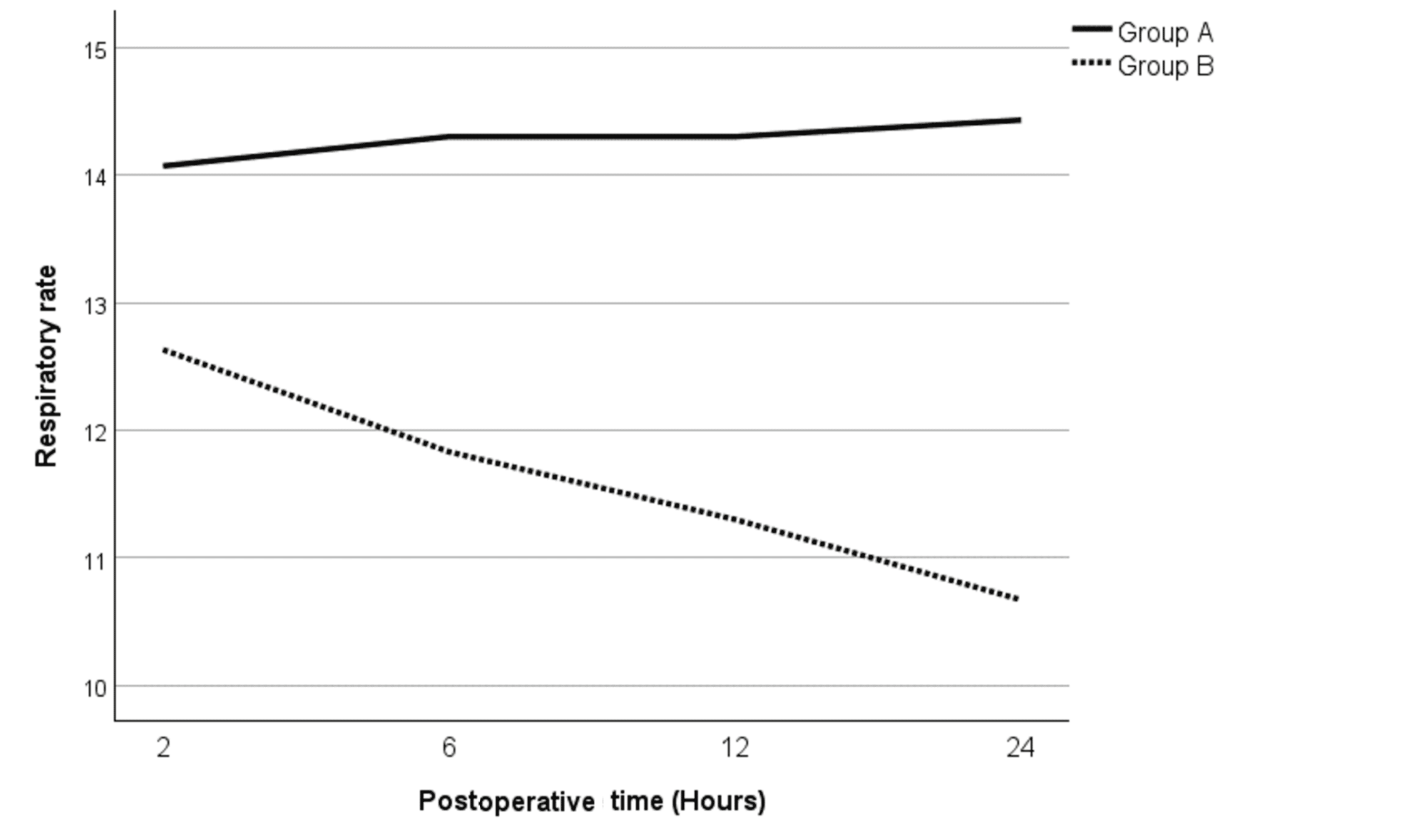
Mean respiratory rate for Groups A and B Test used: independent-sample t test. p ≤ 0.05 considered significant

Patients in both groups remained vitally stable and did not require any significant intervention. Mean arterial pressures (MAPs) and heart rates of the patients in both groups were comparable without any difference. The oxygen saturation in Group B was marginally lower than Group A throughout the study period (Figure 3). The respiratory rate showed a similar pattern with a slightly lower respiratory rate in patients in Group B as compared to Group A (Figure 4). However, both of these differences were not statistically significant.

Other secondary outcomes

**Figure 5:**
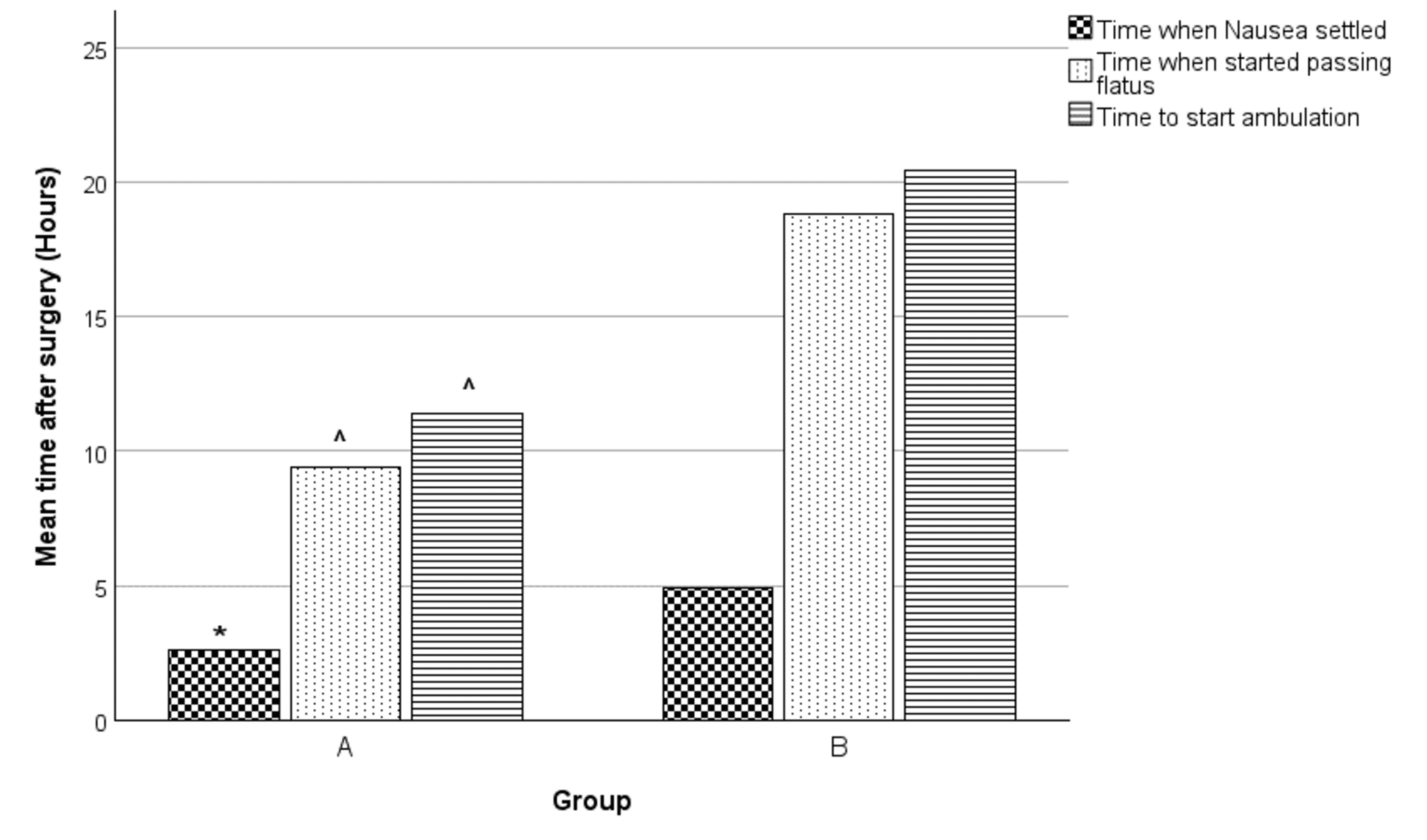
Comparing means of secondary outcomes Test used: independent-sample t test. p ≤ 0.05 considered significant * p < 0.01 when compared to Group B; ^ p < 0.001 when compared to Group B

Postoperative nausea resolved earlier in patients in Group A (2.60 ± 0.37 hours) as compared to Group B (4.93 ± 0.88 hours) (t = -2.45, df = 58, p < 0.01) (Figure 5). Patients in Group A also showed an earlier return of bowel function (as depicted by the passage of flatus) (9.40 ± 0.74 hours) as compared to Group B (18.80 ± 1.10 hours) (t = -7.06, df = 58, p < 0.001) (Figure 5). A similar pattern was seen with regards to time to start ambulation, with patients in Group A beginning ambulation earlier (11.40 ± 0.60 hours) than Group B (20.40 ± 1.02 hours) (t = -7.60, df = 58, p < 0.001) (Figure 5). None of the patients experienced any significant complications and were discharged after 24 hours following the surgery.

## Discussion

Even though open inguinal hernia repair is not considered a major surgery, it can cause moderate-to-severe pain postoperatively, leading to delayed discharges or unplanned readmissions, delayed return to daily activities and work, and poor patient satisfaction [16]. Multiple modalities have been studied to reduce postoperative pain, including pharmacotherapy, loco-regional blocks, and even neurostimulation [16]. However, opioids remain the mainstay for post-procedure analgesia [7].

Opioids modulate pain by acting on µ-receptors in the central nervous system and peripheral tissues [7]. Their good analgesic profile is associated with significant side effects including, but not limited to, nausea, vomiting, ileus, respiratory depression, and dependence [7]. To reduce opioid use, various adjuncts are used clinically, including NSAIDs and nerve blocks, in hopes of limiting the side effects.

In our study, we compared the analgesic efficacy of ketorolac with a weak opioid, tramadol, following open inguinal hernia repair. Pain scores with ketorolac were significantly lower than those with tramadol at all time points. This was in contrast to the results published by Bugada et al., where the authors similarly compared intravenous ketorolac with tramadol and found no difference in analgesic effect [15]. The dose of tramadol used in this study was 100 mg every eight hours (and 50 mg was used if the weight of the patient was < 50 kg). We, however, used 50 mg for all the patients, irrespective of their weight. Despite using a lower dose of tramadol, the pain scores in our study are comparable to those reported by Bugada et al.. In another study by McEvoy et al., the authors compared oral diclofenac sodium with morphine sulfate postoperatively in inguinal hernia day case surgeries and reported improved pain control with diclofenac when compared to morphine [17]. This shows that in simple surgeries like open inguinal hernia repair, the analgesic effects of NSAIDs, when used in isolation, are similar to (if not better than) opioids.

Postoperative nausea, return of bowel function, and ability to ambulate are all factors that affect the length of hospital stay and overall cost. In this study, there was an increased duration of nausea in patients receiving tramadol when compared to those receiving ketorolac. A similar pattern was seen with the time for the return of bowel function (as depicted by the time patients started to pass flatus), with earlier bowel function return seen in the ketorolac group vs. the tramadol group. Similar findings were reported by Bugada et al., where these side effects were more in the tramadol group but not statistically significant [15]. In our study, subjects in the NSAID group also started ambulating earlier as compared to the opioid group. This could be explained by the fact that the pain control was better in the ketorolac group, allowing patients to walk earlier. Early ambulation also helps in the early return of bowel function [18]. On the other hand, nausea and ileus are well-documented side effects of opioid administration [8,9,19], which can be potentiated in the postoperative period, as seen in our patients.

Patients in both groups did not show any difference in the vital signs; however, there was a slight preponderance of respiratory depression in the tramadol group. Even though respiratory rate and oxygen saturation were lower in the tramadol group, this difference was not statistically significant. Opioids cause respiratory depression by acting on the µ-receptors specifically in the pons, which regulate the respiratory rhythm [20]. Tramadol is considered a weak opioid with a relatively safer side effect profile [21]. Risk factors associated with acute respiratory depression in patients using tramadol include the pediatric population, drug abusers, and simultaneous use of other drugs that cause respiratory depression, including benzodiazepines and antidepressants [21]. As our study population did not have these risk factors, severe respiratory depression was not encountered in our patients. However, note should be taken that despite the absence of risk factors, tramadol still showed a tendency to depress respiratory rate and decrease oxygen saturation when compared to NSAIDs.

Similarly, NSAID-induced gastropathy is also well documented in the literature [22,23]; however, it was not seen in our study group. This could be explained due to the fact that all patients received one dose of PPI perioperatively [23]. Also, the duration for which patients received both of these drugs was not sufficient to cause the side effects well documented in the literature [8,9,11,17].

There are a few limitations to this study. Most importantly, patients were only monitored till the time of discharge, which was postoperative day 1. A longer follow-up is required to further elucidate the efficacy of one analgesic modality over the other, along with the side effect profile. Even though our primary outcome was pain score, we did not ask the patients' overall satisfaction with regard to the procedure and pain control at the time of discharge, which is another important measure of the efficacy of the analgesic modality used.

## Conclusions

According to the authors, for open inguinal hernia mesh repair, intravenous ketorolac has a better analgesic profile and fewer side effects when compared to intravenous tramadol. However, neither of these drugs showed any significant complications and are safe at the doses used in this study. Further research needs to be conducted to look at the long-term analgesic effects, along with the efficacy of these drugs in other major surgeries, to further elucidate their effectiveness.
